# A sociodemographic and clinical characterization of Gambling Dual Disorder (GDD) in an italian, cross-sectional multicenter study

**DOI:** 10.1192/j.eurpsy.2026.10441

**Published:** 2026-07-31

**Authors:** R. Leuzzi, N. Girone, C. Ciliberti, G. Delli Zotti, T. Raba, M. A. Colletti, M. Cocchi, F. Ferrero, M. Leonardi, M. Turco, L. Longo, G. Zita, M. I. Fiocchi, B. Benatti, M. Percudani, B. Dell’Osso

**Affiliations:** 1 Department of Biomedical and Clinical Sciences Luigi Sacco, University of Milan; 2 Department of Mental Health and Addiction Services, Niguarda Hospital; 3 ”Aldo Ravelli” Center for Neurotechnology and Brain Therapeutic, University of Milan, Milan, Italy; 4 Department of Psychiatry and Behavioral Sciences, Bipolar Disorders Clinic, Stanford University, California, Standard, CA, United States

## Abstract

**Introduction:**

Gambling Dual Disorder (GDD) has been proposed as a clinical construct to define the co-occurrence of Gambling Disorder (GD) with other psychiatric conditions. Despite the presence of preliminary evidence for this model, limited data are available.

**Objectives:**

The aim of this multicenter, cross-sectional Italian study is to further characterize GDD by comparing individuals with GD only and those with co-occurring psychiatric disorders.

**Methods:**

The sample consists of 227 outpatients diagnosed with GD recruited from two specialized psychiatric centers in Milan: the ASST Grande Ospedale Metropolitano “Niguarda” and the ASST FatebeneFratelli Sacco University Hospital. Patients were divided into two subgroups: GD-only and GDD (i.e., GD plus at least 1 additional psychiatric disorder). Sociodemographic and clinical variables, including the distribution of lifetime comorbidities, were collected. Psychometric questionnaires, e.g. the Canadian Problem Gambling Index (CPGI), the South Oaks Gambling Screen (SOGS) and the Gambling Attitudes and Beliefs (GABS) assessed gambling severity and core psychopathological dimensions. The Barratt Impulsiveness Scale-11 (BIS-11) and the Difficulties in Emotion Regulation Scale (DERS) assessed impulsivity and emotion regulation difficulties, respectively. This study was conducted in accordance with the ethical standards of the relevant national and institutional committees on human experimentation and with the Helsinki Declaration of 1975, as revised in 2008 (PMC2566407).

**Results:**

137 patients (60.4% of the total sample) had criteria for GDD. If compared to the GD-only group (n=90, 39,6% of the total sample), GDD patients were significantly older (44.35 ± 14.85 vs. 36.76 ± 15.03 years; *p*<.05) and reported a later onset of gambling problematics (33.16 ± 13.13 vs. 26.03 ± 11.58 years; *p* < .005). Compared to GD subgroup, the GDD subgroup also displayed higher rates of lifetime suicidal ideation (44.7% vs 18.7%; p<.05) and suicide attempts (6.8% vs 0%; p<.05), along with greater emotional dysregulation in DERS score (102.64 ± 25.23 vs 92.43 ± 25.90; p<.05). Among psychiatric comorbidities, personality disorders (37.2%) and major depressive disorder (28.4%) were the most frequent.

**Conclusions:**

The findings confirm that psychiatric comorbidities have a high prevalence among patients with GD, showing associations with distinct sociodemographic and clinical features. This study supports the increasing clinical relevance of the GDD model, highlighting the importance of integrating it into clinical practice in order to achieve a greater diagnostic accuracy, to improve therapeutic strategies, and to guide future research in the understanding and management of gambling-related disorders.

**Disclosure of Interest:**

None Declared

